# Improved Succinate Production by Metabolic Engineering

**DOI:** 10.1155/2013/538790

**Published:** 2013-04-18

**Authors:** Ke-Ke Cheng, Gen-Yu Wang, Jing Zeng, Jian-An Zhang

**Affiliations:** ^1^Institute of Nuclear and New Energy Technology, Tsinghua University, Beijing 100084, China; ^2^Institute of Applied Chemistry, Department of Chemical Engineering, Tsinghua University, Beijing 100084, China

## Abstract

Succinate is a promising chemical which has wide applications and can be produced by biological route. The history of the biosuccinate production shows that the joint effort of different metabolic engineering approaches brings successful results. In order to enhance the succinate production, multiple metabolical strategies have been sought. In this review, different overproducers for succinate production, including natural succinate overproducers and metabolic engineered overproducers, are examined and the metabolic engineering strategies and performances are discussed. Modification of the mechanism of substrate transportation, knocking-out genes responsible for by-products accumulation, overexpression of the genes directly involved in the pathway, and improvement of internal NADH and ATP formation are some of the strategies applied. Combination of the appropriate genes from homologous and heterologous hosts, extension of substrate, integrated production of succinate, and other high-value-added products are expected to bring a desired objective of producing succinate from renewable resources economically and efficiently.

## 1. Introduction

Succinate and its desirable properties have been known for a long time. Succinate can be used as an important C4 building-block chemical, and its demand is sharply increasing since new applications of this chemical compound are reported in many publications. Of particular interest is that it can be used for 1,4-butanediol synthesis and further as a monomer in a polycondensation reaction yielding biodegradable poly(butylene succinate). 1,4-Butanediol-based polymers have better properties and greater stability in comparison to polymers produced from 1,2-propanediol or ethylene glycol. Further, CO_2_ is assimilated during the succinate biosynthesis which can be considered as an environmental advantage. The commercialization of the polymer produced from biobased succinate by some multinational corporations, such as BioAmber, BASF, Myriant, Mitsubishi, and DuPont, showed that the aim of biobased bulk chemicals is feasible [1–4]. 

Biotechnological processes are particularly attractive since microorganisms usually utilize renewable feedstock and only produce few toxic by-products. However, there are limitations of microbial production like limited yields, concentrations and productivities, difficulties in the product recovery from the broth, or the need of pretreatment of most of the raw substrates. These limitations can be significantly improved through application of metabolic engineering. New strategies for succinate production improvement by metabolic engineering are frequently reported. Metabolic engineering of the microbial succinate production covers natural succinate overproducers (like* Actinobacillus succinogenes*, *Anaerobiospirillum succiniciproducens*, and *Mannheimia succiniciproducens*) as well as metabolic engineered overproducers like* Escherichia coli*, *Corynebacterium glutamicum,* and *Saccharomyces cerevisiae*. Genes from homologous and heterologous hosts were often used in combination to complete the pathway [1, 2]. In this review, different overproducers for succinate production are examined, and the metabolic engineering strategies and performances are discussed. Finally, strategies for successful commercialization of succinate production by improvement of metabolic engineered are proposed.

## 2. Succinate Formation Pathway

Besides as an intermediate of the tricarboxylic acid (TCA) cycle, succinate can also be a fermentation end product when sugar or glycerol is used as a carbon source. There are three pathways for succinate formation including the reductive branch of the TCA cycle, the glyoxylate pathway, and the oxidative TCA cycle [5–10].

**Figure 1:**
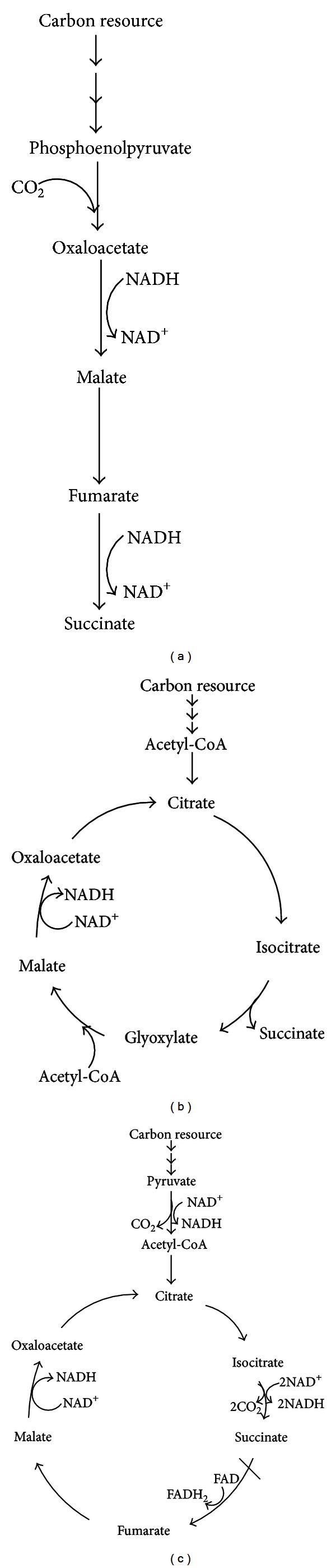
Succinate production pathway from (a) the reductive branch of the TCA cycle. Succinate accumulates derived from phosphoenolpyruvate, via some intermediate, including oxaloacetate, malate, and fumarate. (b) The glyoxylate pathway. The glyoxylate pathway operates as a cycle to convert 2 mol acetyl CoA to 1 mol succinate. (c) The oxidative TCA cycle. This pathway converts acetyl-CoA to citrate, isocitrate, and succinate and subsequently converted to fumarate by succinate dehydrogenase. Under aerobic conditions, the production of succinate is not naturally possible, and to realize succinate accumulation under aerobic condition, inactivation of *sdhA* gene to block the conversion of succinate to fumarate in TCA cycle is necessary.

Under anaerobic conditions, succinate is the H-acceptor instead of oxygen, and therefore the reductive branch of the TCA cycle is used. Succinate accumulates derived from phosphoenolpyruvate (PEP), via some intermediate compounds of TCA reductive branch, including oxaloacetate (OAA), malate, and fumarate (Figure 1(a)). The pathway converts oxaloacetate to malate, fumarate, and then succinate, which requires 2 moles of NADH per mole of succinate produced [5–8]. The equation of anaerobic pathway is

(1) $$\begin{array}{c} \text{PEP}+{\text{CO}}_{2}+2\text{NADH}\to \text{succinate}+2{\text{NAD}}^{+} \end{array}$$

The maximum possible succinate yield based solely on a carbon balance is 2 mol mol^−1^ glucose when all the succinate is formed via the anaerobic pathway. One major obstacle to obtain high succinate yield through the anaerobic pathway is NADH limitation. This is because 1 mole glucose can provide only 2 moles of NADH through the glycolytic pathway. Therefore, the molar yield of succinate is limited to 1 mol mol^−1^ glucose assuming that all the carbon flux will go only through the anaerobic fermentative pathway.

Another potential biosynthetic route for succinate is through the glyoxylate pathway, which is an anaplerotic reaction to fill up the molecule pool of the TCA. The glyoxylate cycle is essentially active under aerobic conditions upon adaptation to growth on acetate (Figure 1(b)). The glyoxylate pathway operates as a cycle to convert 2 mol acetyl CoA to 1 mol succinate [9]. The equation of glyoxylate pathway is:

(2) 2Acetyl  CoA+2H2O+NAD+  →Succinate+2CoASH+NADH+2H+

Since the conversion of glucose to succinate via glyoxylate pathway generates NADH, this route alone is not sufficient to balance the electrons. However, during anaerobic culture and in the absence of an additional electron donor, activated glyoxylate pathway will provide extra NADH to anaerobic fermentative pathway and benefit to achieve higher succinate yield.

Succinate can also be formed from acetyl-CoA generated from pyruvate via oxidative TCA cycle under aerobic conditions [10]. This pathway converts acetyl-CoA to citrate, isocitrate, and succinate which is subsequently converted to fumarate by succinate dehydrogenase. Under aerobic conditions, the production of succinate is not naturally possible since it is only an intermediate of the TCA cycle. To realize succinate accumulation under aerobic condition, inactivation of *sdhA* gene to block the conversion of succinate to fumarate in TCA cycle is necessary (Figure 1(c)). The equation of aerobic pathway is

(3) 2Pyrurate+2H2O+3NAD+  →Succinate+3NADH+2CO2

## 3. Debottlenecking of the Succinate Pathway

The yield of succinate in anaerobic culture from sugar or other feedstock is strongly decided by available NADH produced in the glycolysis route which results in by-products' accumulations [11–13]. The by-product formation is caused by the redox balance from substrate to product. By-product accumulations result in a substrate loss as well as usual product inhibition, such as formate, acetate, and lactate accumulation, whose undissociated form will be harmful for biomass formation and substrate consumption. Also, succinate itself harms the microorganisms in the same way like other weak organic acids. Since the dissociation constants of these weak organic acids and the resistance of microorganisms to these acids inhibition are different, genetic engineering tools can also be used to manipulate the metabolic pathways so that target product pathway can be strengthened or by-product pathways can be selectively eliminated [14].

Deletion of the one of undesired metabolites' pathways will divert carbon resource to other metabolites. However, in some case, the desired product is not improved due to unreasonable metabolic flux redistribution and new undesired metabolite's accumulation. Furthermore, elimination of some by-products will break original intracellular redox balance. So, a reasonable design of metabolic net is needed before genetic manipulations so that the redox or ATP is kept at good balance and the production of desired product is enhanced.

## 4. Metabolic Engineering of the Succinate Producer

*A. succinogenes*, *A. succiniciproducens*, *M. succiniciproducens* are well-known natural overproducers and there were some efforts to improve the yield. *E. coli*, *C. glutamicum,* and *S. cerevisiae *are not natural overproducers, and therefore completely genetic engineered pathway was sought for acquired ability to form succinate. Succinate production using different bacteria species in terms of performances and engineering strategies is compared in Table 1.

*A. succinogenes* mutant strain FZ-6, with pyruvate formate lyase and formate dehydrogenase deletion, did not show improved succinate production. Only when electrically reduced neutral red or hydrogen was fed as the electron donor, the mutant can use fumarate alone for succinate production [34]. Major metabolic pathways in *M. succiniciproducens* resulting in acetate, formate, and lactate accumulation were successfully deleted by disrupting the *ldhA*, *pflB*, *pta*, and *ackA* genes. A modified strain LPK7 was developed to excrete 13.4 g L^−1^ succinate using 20 g L^−1^ glucose with little or no by-product accumulation. In fed-batch fermentation by occasional glucose feeding,* M. succiniciproducens* LPK7 produced 52.4 g L^−1^ succinate, giving a yield of 0.76 g g^−1^ glucose and a productivity of 1.8 g L^−1^ h^−1^ [15].

### 4.1. Escherichia coli

**Figure 2:**
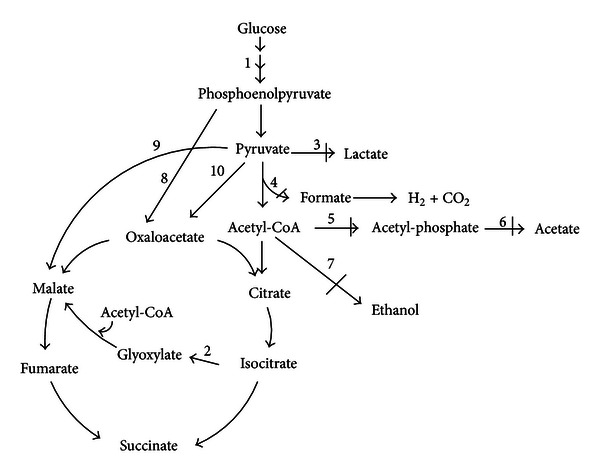
Fermentation of glucose to succinate by genetically engineered central anaerobic metabolic pathway. Bold vertical bar means that the relative gene was inactivated. Bold arrows show overexpression. (1) The PEP-dependent glucose uptake system is replaced with ATP-dependent phosphorylation, (2) the activation of the glyoxylate pathway, (3) the knockouts of lactate (lactate dehydrogenase), (4) the knockouts of formate pathway (pyruvate formate-lyase), (5) and (6) the knockouts of acetate pathway (acetate kinase, phosphate acetyltransferase), (7) the knockouts of ethanol pathway (alcohol dehydrogenase), (8) overexpressed phosphoenolpyruvate carboxylase, (9) overexpressed malic enzyme, (10) overexpressed pyruvate carboxylase.

Due to plentiful of genetic tools available, fast cell growth, and simple culture medium, *E. coli* has turned to one of the most wholly studied systems for succinate production. Strategies in metabolic engineering of *E. coli* can be classified as four main methods: improvement of substrate or product transportation, enhancement of pathways directly involved in the succinate production, deletion of pathways involved in by-product accumulation, and their combinations (Figure 2). These methods have been studied in many reports and some high efficient succinate producers have been constructed [35–38]. 

## 5. Improvement of Substrate or Product Transportation

A fundamental change imposed on *E. coli* is the elimination of glucose transport by the phosphotransferase system (PTS). This modification addresses the limitation that glucose phosphorylation in *E. coli* is largely PEP dependent. If PEP is generated solely via the Embden-Meyerhof-Parnas pathway, this constraint imposes an artificial ceiling yield of 1 mol mol^−1^ glucose in succinate production. Although alternate routes for the generation of PEP exist, glucose phosphorylation is energetically more efficient if the PEP-dependent system is replaced with ATP-dependent phosphorylation. Further, more PEP are reserved and used for the succinate formation route. Some successful constructed *E. coli* strains for succinate production, such as AFP111 and KJ060, mainly rely on glucokinase for glucose uptake, which has been confirmed by metabolic flux and enzymatic analysis [35, 39]. 

Succinate export in *E. coli* is normally active under anaerobic conditions and import only under aerobic conditions. The dicarboxylic acid transport system of *E. coli* was modified by Beauprez et al. to enhance production of succinate. The engineering comprised the elimination of succinate uptake and the enhancement of succinate output. The gene responsible for succinate import, *dctA*, was knocked out, and the gene coding for succinate export, *dcuC*, was overexpressed with a constitutive artificial promoter. The combination of altered import (*ΔdctA*) and export (*ΔFNR-pro37-dcuC*) increased the specific production rate by about 55% and the yield by approximately 53% [40].

## 6. Enhancement of Pathways Directly Involved in the Succinate Production

Overexpression of genes directly involved in the succinate production pathway, including PEP carboxylase, PEP carboxykinase, pyruvate carboxylase, and malic enzyme, was reported in a lot of publications. In a study by Millard et al., succinate production using* E. coli* JCL 1208 increased from 3 g L^−1^ to 10.7 g L^−1^ by overexpressing native PEP carboxylase [16]. However, overexpression of PEP carboxykinase did not affect succinate production. Due to the intimate role that PEP is needed as substrate for glucose transport by the phosphotransferase system in wild-type* E. coli*, the consequences of overexpressing PEP carboxylase are decreasing rate of glucose uptake and organic acid excretion [41]. Another method is to improve pyruvate to the succinate synthesis route through the expression of pyruvate carboxylase, an enzyme which can convert pyruvate to oxaloacetate but not contained in *E. coli*. A wild-type *E. coli* strain MG1655 transformed with vector pUC18-pyc, which contained the gene encoding for *Rhizobium etli* pyruvate carboxylase, led to a succinate formation of 1.77 g L^−1^, corresponding to a 50% increase in succinate concentration using the parent strain. The increased succinate was due to decreased lactate formation, whose final concentration decreased from 2.33 g L^−1^ to 1.88 g L^−1^. The expression of pyruvate carboxylase had no effect on the glucose uptake, but decreased the rate of cell growth [17].

## 7. Deletion of Pathways Involved in By-Product Accumulation

Only shift of carbon flux to succinate route is not enough to control the formation of other undesired metabolites. As a consequence, genetically modified strains without lactate and formate forming routes are developed to improve succinate fermentation. Mutant of *E. coli* LS1, which only lacked lactate dehydrogenase, had no effect on anaerobic biomass formation. However, *E. coli* NZN111, deficient in both the pyruvate-formate lyase and lactate dehydrogenase genes, respectively, gave few biomass formations on glucose. NZN111 accumulated 0.18–0.26 g L^−1^ pyruvate before metabolism ceased, even when supplied with acetate for biosynthetic needs. However, when transformed with the *mdh* gene encoding NAD^+^-dependent malic enzyme and cultured from aerobic environment converting to anaerobic environment gradually (by metabolically depleting oxygen initially present in a sealed culture tube), *E. coli* NZN111 was allowed to consume all the glucose. 12.8 g L^−1^succinate was produced as one of the major metabolites [18]. Analogously, when the gene encoding malic enzyme from *Ascaris suum *was transformed into NZN111, succinate yield was 0.39 g g^−1^ and productivity was 0.29 g L^−1^ h^−1^ [18]. 

## 8. Optimization of the Succinate Yield by a Combination of Gene Operations

Donnelly et al. screened a spontaneous chromosomal mutation in NZN111, and this mutation was named AFP111, which can grow on glucose in anaerobic environment. A succinate yield of 0.7 g g^−1^ was obtained using AFP111 in anaerobic fermentations under 5% H_2_-95% CO_2_ flow, and the molar ratio between succinate and acetate is 1.97 [19]. Further, if AFP111 first grew under aerobic conditions for biomass formation and then was shifted to anaerobic fermentation with CO_2_ aeration (dual-phase fermentation), higher succinate yield (0.96 g g^−1^) with a productivity of 1.21 g L^−1^ h^−1^ was obtained [20, 21]. In Chatterjee et al.'s report, the difference between NZN111 and AFP111 was found to be the PTS. Because of the PTS mutation, AFP111 mainly relied on glucokinase for glucose uptake. No matter anaerobic or aerobic environment for cell growth, AFP111 exhibited obviously higher glucokinase activity than that of NZN111. Compared with the wild-type parent strain W1458, AFP111 also showed a lower glucose uptake [39]. According to Vemuri et al., the routes for PEP conversion to succinate also varied between NZN111 and AFP111. The key glyoxylate shunt enzyme, isocitrate lyase, was not present with both NZN111 and AFP111 grown under anaerobic environment but was detected after 8 h of aerobic culture, and NZN111 exhibited 4-fold higher isocitrate lyase activity than AFP111 [20]. Because the two strains have different modes of glucose uptake and different isocitrate lyase activity level, the distribution of end products in the two strains is different. Further, they concluded that the maximum theoretical succinate yield based on the necessary redox balance is 1.12 g g^−1^ glucose (Table 2). For obtaining the maximal succinate yield, the molar ratio of the carbon flux from fumarate to succinate to the carbon flux from isocitrate to succinate must be 5.0. Insufficient carbon flux through the PEP-to-fumarate branch or elevated carbon flux through the glyoxylate shunt lowers the observed yield. Achieving the optimal ratio of fluxes in the two pathways involved in anaerobic succinate accumulation requires a concomitant balance in the activities of the participating enzymes, which have been expressed during aerobic growth. The global regulation of aerobic and anaerobic pathways is further complicated by the presence of different isozymes. Based on carbon flux analysis, this group transformed AFP111 with the *pyc* gene (encode *Rhizobium etli* pyruvate carboxylase) to provide metabolic flexibility at the pyruvate node. A two-stage aeration strategy (firstly aerobic fermentations with *K*_*L*_ *a* 52 h^−1^; when dissolved oxygen concentration decreases to 90% of initial concentration, shift to anaerobic fermentation with oxygen-free CO_2_ sparged) was applied in the fermentation for higher succinate yield and productivity. Using this strategy, a final 99.2 g L^−1^ succinate concentration with a yield of 1.1 g g^−1^ and a productivity of 1.3 g L^−1^ h^−1^ was obtained [21].

Serial publications on modification of *E. coli* MG1655 strain for improved succinate production were reported by San's group. Firstly, SBS110MG (pHL413) was created from an *adhE*, *ldhA* mutant strain of *E. coli*, SBS110MG, harboring plasmid pHL413, which encoded the *Lactococcus lactis* pyruvate carboxylase. After 48 h fermentation using this strain, 15.6 g succinate L^−1^ was produced with a yield of 0.85 g g^−1^ [22]. *E. coli* SBS550MG (pHL413) was further developed by deleting *adhE*, *ldhA* and *ack-pta* routes and by activating the glyoxylate routes by deactivation of *iclR*. By a repeated glucose feeding, SBS550MG (pHL413) produced 40 g L^−1^ succinate with a yield of 1.05 g g^−1^ glucose. They found that the best distribution ratio for the highest succinate yield was the fractional partition of OAA to glyoxylate of 0.32 and 0.68 to the malate [24]. In addition, this group studied the effect of overexpressing a NADH insensitive citrate synthase from *B. subtilis* on succinate fermentation. They found that this change had no effect on succinate yield but affected formate and acetate distribution. Furthermore, Sánchez et al. tried to place an *arcA* mutation within the host, SBS550MG, leading to a strain designated as SBS660MG. The phosphorylated *arcA* is a dual transcriptional regulator of aerobic respiration control, which also suppresses transcription of the *aceBAK*. Deactivation of *arcA* could further improve the transcription of *aceBAK* and hence led to a more efficient glyoxylate pathway. Unfortunately, SBS660MG did not improve the succinate yield and it reduced glucose consumption by 80%. It should be pointed out that succinate production experiments in the previous description were carried out in a two-stage aeration culture in bioreactors where the first stage was aerobic for cell growth followed by an anaerobic stage for succinate accumulation and the initial inoculum was very large (initial dry cell weight 5.6 g L^−1^). Aeration condition during the cell growth stage has great impact on anaerobic succinate accumulation using *E. coli* SBS550MG (pHL413) [23]. Martínez et al. found that a microaerobic environment was more suitable for succinate production. Compared with microaerobic environment, the high aeration experiment led to more pyruvate accumulation, which correlated with a lower *pflAB* expression during the transition time and a lower flux towards acetyl-CoA during the anaerobic stage. The improvement in glyoxylate shunt-related genes expression (*aceA*, *aceB*, *acnA*, *acnB*) during the transition time, anaerobic stage, or both increased succinate yield in microaerobic environment [42]. 

Because intracellular acetyl-CoA and CoA concentrations can be increased by overexpression of *E. coli* pantothenate kinase (PANK) and acetyl-CoA is a promising activator for PEP carboxylase (PEPC) and pyruvate carboxylase (PYC), Lin et al. constructed *E. coli* GJT (pHL333, pRV380) and GJT (pTrc99A, pDHK29) by coexpressing of PANK and PEPC, and PANK and PYC, respectively. They found that coexpression of PANK and PEPC, or PANK and PYC, did enhance succinate accumulation, but lactate production decreased significantly [25]. In a subsequent report, GJT (pHL333, pHL413) coexpression of PEPC and PYC contributed to 2.05 g L^−1^ succinate accumulation. When both the acetate (*ackA*-*pta*) and lactate pathways (*ldhA*) were deactivated in YBS132 (pHL333, pHL413), succinate concentration and yield increased by 67% and 76%, respectively, compared with the control strain GJT (pHL333, pHL413) (3.4 g L^−1^ versus 2.1 g L^−1^, 0.2 g g^−1^ versus 0.11 g g^−1^). No lactate was detected and acetate accumulation reduced by 76% [27]. For higher cell growth, faster substrate consumption, and product accumulation, mutation in the tricarboxylic acid cycle (*sdhAB*, *icd*, *iclR*) and acetate pathways (*poxB*, *ackA*-*pta*) of *E. coli* HL51276k was developed to construct the glyoxylate cycle for producing succinate aerobically. After 80 h fermentation, succinate production reached 4.61 g L^−1^ with a yield of 0.43 g g^−1^ glucose. The substantial accumulations of pyruvate and TCA cycle C_6_ intermediates were observed during aerobic fermentation which hindered achieving the maximum succinate theoretical yield. PEPC from *Sorghum* was overexpressed in the strain HL51276k [26]. At approximately 58 h, HL51276k (pKK313) produced 8 g L^−1^ succinate with a succinate yield of 0.72 g g^−1^ glucose. Overexpression of PEPC was also effective in decreasing pyruvate accumulation (30 mM in HL51276k (pKK313) versus 48 mM in HL51276k). Further, they found that an overexpression of PEPC and deactivation of* ptsG* combined strategy was the most efficient in decreasing pyruvate accumulation.

By a combination of gene deletions on *E. coli* ATCC 8739 and multiple generations of growth-based selection, a high succinate producing strain KJ060 (*ldhA*, *adhE*,* ackA*, *focA*, *pflB*) was obtained, producing 86.6 g L^−1^ of succinate with a yields of 0.92 g g^−1^ glucose and a productivity of 0.9 g L^−1^ h^−1^ [28]. After selection, PEP carboxylase was replaced by the gluconeogenic PEP carboxykinase through spontaneous mutation to the major carboxylation pathway for succinate formation. The PEP-dependent phosphotransferase system was deactivated by a spontaneous point mutation and functionally replaced by the GalP permease and glucokinase. By these improvements, the net ATP molar yield during succinate formation was increased to 2.0 ATP per glucose. This improved *E. coli* pathway is similar to the pathway of native succinate producing rumen bacteria. For higher yield and lower by-product formation, new generation KJ134 (*ΔldhA ΔadhE ΔfocA-pflB ΔmgsA ΔpoxB ΔtdcDE ΔcitF ΔaspC ΔsfcA Δpta-ackA*) was constructed, producing 71.6 g L^−1^ succinate with a yield of 1 g g^−1^ glucose and a productivity of 0.75 g L^−1^ h^−1^ in batch fermentations using mineral salts medium under anaerobic environment. Compared with KJ060, by-product acetate of KJ134 decreases 85% (4.4 g L^−1^ versus 29.5 g L^−1^), which is very useful in product recovery for the commercial production of succinate.

### 8.1. Corynebacterium glutamicum

*C. glutamicum* is a fast-growing, nonmotile, gram-positive microorganism with a long history in the microbial fermentation industry for amino acids and nucleic acids. *C. glutamicum*, under oxygen deprivation without growth, produces organic acids such as lactic acid, succinate, and acetic acid from glucose. These features allow for the use of high-density cells, leading to a high volumetric productivity. Some studies have been carried out on *C. glutamicum* with respect to the microbial production of succinate [29, 43].

In order to eliminate lactic acid production, deletion of *ldhA* gene coding for L-lactate dehydrogenase was targeted in *C. glutamicum *R. A lactate-dehydrogenase-(LDH-) deficient mutant was not able to produce lactate, suggesting that this enzyme has no other isozyme. Moreover, overexpression of genes coding for anaplerotic enzymes in the mutant demonstrated that the rate of succinate production of the resultant strain *C. glutamicum ΔldhA*-pCRA717 with enhanced pyruvate carboxylase activity was 1.5-fold higher than that of parental mutant strain. Using this strain at a dry cell weight of 50 g L^−1^ under oxygen deprivation, succinate was produced efficiently with intermittent addition of sodium bicarbonate and glucose. The succinate production rate and yield depended on medium bicarbonate concentration rather than glucose concentration. Succinate concentration reached 146 g L^−1^ with a yield of 0.92 g g^−1^ and a productivity of 3.2 g L^−1^ h^−1^ [29].

### 8.2. Saccharomyces cerevisiae

*S. cerevisiae* is genome sequenced, genetically and physiologically well characterized, and can produce organic acids even at the low pH that facilitates downstream. Many tools for genetic improvement are established. These features make *S. cerevisiae* suitable for the biotechnological production of succinate. Due to this fact, attempts to engineer *S. cerevisiae* for the succinate production were made.

Using ^13^C flux analysis, Camarasa et al. found that during anaerobic glucose fermentation by* S. cerevisiae*, the reductive branch generating succinate via fumarate reductase operates independently of the nitrogen source. This pathway is the main source of succinate during fermentation, unless glutamate is the sole nitrogen source, in which case the oxidative decarboxylation of 2-oxoglutarate generates additional succinate [44]. 

*S. cerevisiae* is a well-known glycerol and ethanol producer. Therefore, with respect to this microorganism, synthesis of succinate must limit the formation of glycerol and ethanol. In a patent issued by Verwaal et al., *S. cerevisiae* RWB064 with a deletion of the genes alcohol dehydrogenase 1 and 2 and the gene glycerol-3-phosphate dehydrogenase 1 was used for the parental strain. The genes PEP carboxykinase from *A. succinogenes*, NADH-dependent fumarate reductase from *Trypanosoma brucei*, fumarase from *Rhizopus oryzae*, malate dehydrogenase from *S. cerevisiae,* and malic acid transporter protein from *Schizosaccharomyces pombe* were overexpressed. The recombinant SUC-200 produces 34.5 g L^−1^succinate, and the main by-products include 4.5 g L^−1^ ethanol, 7.7 g L^−1^ glycerol, and 7.8 g L^−1^ malate. Further improvement of the recombinant SUC-297 included overexpression of pyruvate carboxylase from *S. cerevisiae*. Succinate formation increases to 43 g L^−1^ and no malate accumulates. Since succinate, glycerol, and ethanol have different volatility, they can be easily purified [30].

Arikawa et al. reported an improved succinate production using sake yeast strains with some TCA cycle genes deletions [45]. Compared with the wild-type strain, succinate production was increased up to 2.7-fold in a strain with simultaneous disruption of a subunit of succinate dehydrogenase (SDH1) and fumarase (FUM1) under aerobic conditions. The single deletion of gene SDH1 led to a 1.6-fold increase of succinate. These enhancements were not observed under strictly anaerobic or sake brewing conditions. Absence or limitation of oxygen resulted in decreased succinate production in *sdh1* and/or *fum1* deletion strains [31]. In another study on sake yeast strains, the deletion of genes encoding for succinate dehydrogenase subunits (SDH1, SDH2, SDH3, and SDH4) also resulted in increased succinate production only under aerobic conditions [46].

In order to redirect the carbon flux into the glyoxylate cycle and to improve succinate accumulation, the disruption of isocitrate dehydrogenase activity is also part of a metabolic strategy for succinate production in the oxidative branch. Succinate production reduced to approximate half in comparison with the parental strain was observed for yeast strains with disruptions of isocitrate dehydrogenase subunits (IDH1 or IDH2) under sake brewing conditions [47]. The constructed yeast strains with disruptions in the TCA cycle after the intermediates isocitrate and succinate by four gene deletions (*Δsdh1 Δsdh2 Δidh1 Δidp1*) produce 3.62 g L^−1^ succinate at a yield of 0.072 g g^−1^ glucose and do not exhibit serious growth constraints on glucose. The main by-products are 14 g L^−1^ ethanol, 3.8 g L^−1^ glycerol, and 0.8 g L^−1^ acetate [33]. With the aim to reduce fermentation by-products and to promote respiratory metabolism by shifting the fermentative/oxidative balance, the constitutive overexpressions of the SAK1 and HAP4 genes in *S. cerevisiae* were carried out also by Raab et al. Sak1p is one of three kinases responsible for the phosphorylation, and thereby the activation of the Snf1p complex, which plays a major role in the glucose derepression cascade. Hap4p is the activator subunit of the Hap2/3/4/5 transcriptional complex. Hap4p overexpression resulted in increased growth rates and biomass formation, while levels of ethanol and glycerol were decreased. The *sdh2 *deletion strain with *SAK1 *and *HAP4 *overexpression produced 8.5 g L^−1^ succinate with a yield of 0.26 mol mol^−1^ glucose. No glycerol formation was found, and the ethanol produced after 24 h fermentation was consumed for acetate formation [48].

A multigene deletion *S. cerevisiae* 8D was constructed by Otero et al., and directed evolution was used to select a succinate producing mutant. The metabolic engineering strategy included deletion of the primary succinate consuming reaction (succinate to fumarate) and interruption of glycolysis derived serine by deletion of 3-phosphoglycerate dehydrogenase. The remodeling of central carbon flux towards succinate minimized the conversion of succinate to fumarate and forced the biomass-required amino acids L-glycine and L-serine to be produced from glyoxylate pools. The mutant strain 8D with isocitrate lyase overexpression represented a 30-fold improvement in succinate concentration and a 43-fold improvement in succinate yield on biomass, with only a 2.8-fold decrease in the specific growth rate compared to the reference strain [32].

## 9. Final Remarks

At present, some succinate productions using *A. succinogenes* and *A. succiniciproducens* are studied under environments absolutely free of oxygen for cultivation. Anaerobic fermentation is preferred because of its lower capital and operational costs when compared to aerobic fermentations. However, their applicability in the industrial process is limited to some extent because of their maximum possible succinate yield (1 mol mol^−1^ glucose). Also, few genetic tools may seriously limit these strains' reconstruction of metabolic engineering. *E. coli* is the ideal host of choice in future due to in-depth knowledge on this species gained over the past decades, high possible succinate yield (1.72 mol mol^−1^ glucose), and general acceptance of their usage in industrial processes. Other metabolic engineered overproducers like *C. glutamicum Δldh*A-pCRA717, which can produce 146 g l^−1^ succinate in a cell recycling fed-batch culture, deserve more attention in the future.

In order to improve the succinate production by metabolic engineering approaches, various strategies have been investigated and applied in different hosts with acquired ability to produce succinate. Multiple studies have been dedicated to overcome current limitations of the process, like introduction of an ATP-dependent glucose transport system, knockout of LDH, ADHE, or ACKA encoding genes to eliminate by-products formation, to favorably change the ATP formation, to restrict accumulation of pyruvate, and so forth. To seek economical and robust biotechnological production of succinate, considerable progress has been made. However, much work needs to be done in order to achieve the desired process efficiency. Better understanding of metabolic background of both native succinate producers and heterologous hosts is essential for further efforts of directed metabolic engineering. Improvements with internal redox balance, efficient overexpression of required enzyme activities, and modification of by-products formation are expected to approach higher product concentration and yield. 

After the great success of succinate production from glucose, it is the time to develop an efficient process from raw glycerol and biomass (like lignocellulosic substrates or agroindustrial waste products). Being more reduced than glucose, each glycerol could be converted to succinate and could maintain redox balance. Thus, the use of glycerol is more favorable for succinate yield. Hydrolysate from biomass is a mixture of sugars containing mainly glucose and xylose. When glucose and xylose are present at the same time, xylose consumption generally does not start until glucose is depleted. Microbial preference for glucose is caused by the regulatory mechanism named carbon catabolite repression. Metabolic engineering approach to eliminate glucose repression of xylose utilization is very important, so that glucose and xylose could be consumed simultaneously to produce succinate [53–60]. Furthermore, to increase the competitiveness of biological succinate, strategies can be efficiently utilized for the coproduction of succinate and other high-value-added products, such as propanediol and succinate, isoamyl acetate and succinate, and polyhydroxybutyrate and succinate (Table 3). This type of fermentation producing two commercial interests at the same fermentation process might be considered for a promising biological production process which will decrease the production cost by sharing the recovery cost and operation cost.

## 10. Conclusion 

Metabolic engineering focuses on the improvement of microbial metabolic capabilities via improving existing pathways and/or introducing new pathways. The metabolic engineering approach has been widely applied to improve biological succinate production. As a consequence, there has been significant progress in optimization of succinate producing machineries, elimination of biochemical reactions competing with succinate production, and the incorporation of nonnative metabolic pathways leading to succinate production. However, the performance of most succinate producers in terms of concentration, productivity, yield, and industrial robustness is still not satisfactory. More studies, like extension of substrate, combination of the appropriate genes from homologous and heterologous hosts, and integrated production of succinate with other high-value-added products, are in progress. 

## Figures and Tables

**Table 1:** Comparison of succinate production using different bacteria species in terms of performance and engineering strategies.

Strains			Succinate production					
	Engineering strategies	Culture methods	Concentration (g L^−1^)	Productivity (g L^−1^ h^−1^)	Yield (g g^−1^)	Biomass	By-product	References
*Mannheimia succiniciproducens *								

LPK7	Deletion of the LDH, PFL, PTA, ACK	Anaerobic, batch	13.4	1.18	0.67	1.5 g L^−1^	Pyruvate, malate	
		Anaerobic, fed-batch	52.4	1.75	0.76	2.5 g L^−1^	Pyruvate, malate	[15]

*Escherichia coli *								

JCL1208 (pCP201)	Overexpressed PEPC	Anaerobic, batch	10.7	0.59	0.29	—	Ethanol, acetate, lactate, formate	[16]
MG1655-*pyc *	Overexpressed PYC	Anaerobic, batch	1.77	0.177	0.177	0.9 g L^−1^	Lactate, formate	[17]
NZN111	Deletion of PFL and LDH. Overexpressed MAE	Anaerobic, batch	12.8	0.29	0.64	—	Acetate	[18]
AFP111	Glucose transport by ATP-dependent phosphorylation. Deletion of PFL and LDH	Anaerobic, batch	12.8	—	0.7	0.16 g L^−1^	Acetate, ethanol	[19]
		Dual phase aeration, fed-batch	—	1.21	0.96	10 (OD600)	Acetate	[20]
AFP111-*pyc *	AFP111 with overexpressed PYC	Dual-phase aeration, fed-batch	99.2	1.31	1.1	—	Pyruvate, formate	[21]
SBS110MG	Deletion of LDH, PFL Overexpressed PYC	Anaerobic, batch	15.6	0.33	0.85	—	Acetate, formate	[22]
SBS550MG (pHL314)	Deletion of ADH, LDH, ICLR, and ACK-PTA Overexpressed PYC	Anaerobic, fed-batch	40	0.42	1.06	17 (OD600)	Formate, acetate	[23]
SBS990MG (pHL314)	Deletion of ADHE, LDHA, ACK-PTA Overexpressed PYC	Anaerobic, batch	15.9	0.64	1.07	9 (OD600)	Formate	[24]
GJT (pHL333, pHL413)	Coexpression of PEPC and pantothenate kinase (PANK)	Anaerobic, batch	2.05	0.085	0.1	—	Lactate	[25]
YBS132 (pHL333, pHL413)	Coexpression of PEPC and PYC Deletion of LDH and ACK-PKA	Anaerobic, batch	3.4	0.14	0.2	—	Acetate, ethanol	[26]
HL51276k	Mutation in the tricarboxylic acid cycle (SDHAB, ICD, ICLR) and acetate pathways (POXB, ACKA-PTA)	Aerobic, batch	4.61	0.06	0.43	—	Pyruvate, acetate	[27]
HL51276k- pepc	HL51276k with overexpressed PEPC	Aerobic, batch	8	0.14	0.72	—	Pyruvate, acetate	[27]
KJ060	Deletion of LDH, ADHE, ACKA, FOCA, PFLB. Glucose transport by ATP-dependent phosphorylation. PEPC was replaced by the gluconeogenic PEPC	Anaerobic, fed-batch	86.6	0.9	0.92	2.2 g L^−1^	Malate, acetate	[8]
KJ134	Deletion of LDH, ADHE, FOCA-PFLB, MGSA, POXB, TDCDE, CITF, ASPC, SFCA, PTA-ACKA. Glucose transport by ATP-dependent phosphorylation. PEPC was replaced by the gluconeogenic PEPC	Anaerobic, fed-batch	71.6	0.75	1	2.3 g L^−1^	Acetate, pyruvate, malate	[28]

*Corynebacterium glutamicum *								

Δ*ldhA*-pCRA717	Deletion of LDHa. Overexpressed PYC	Micro-aerobic, fed-batch with membrane for cell recycling	146	3.17	0.92	60 g L^−1^	Acetate	[29]

*Saccharomyces cerevisiae *								

Suc-200	Deletion of ADH1, ADH2 and GPD1. Overexpressed PCKa, FUMR, FUM, MDH and MAE1	Aerobic, fed-batch	34.5	0.24	—	—	—	[30]
Suc-297	Deletion of ADH1, ADH2 and GPD1 Overexpressed PEPC, NADH-dependent FUMR, FUM, MDH and malic acid transporter protein, PYC	Aerobic, fed-batch	43	0.45	—	—	—	[30]
Kura	Deletion of SDH1 and FUM1	Aerobic, batch	2.32	0.005	0.015	—	Ethanol, malate, lactate	[31]
8D (pRS426T-ICL1-C)	Deletion of SDH and SER3/SER33 Overexpression of ICL1	Aerobic, batch	0.9	0.05 g/g	—	—	—	[32]
*AH22ura3 *Δ*sdh2*Δ*sdh1*Δ*idh1*Δ*idp1 *	Deletion of SDH and IDH	Aerobic, batch	3.62	0.022	0.072	7.0 g L^−1^	Ethanol, glycerol, acetate	[33]

**Table 2:** Comparison of maximum theoretical yield of each pathway in *E. coli*.

Means of culture	Means of glucose intake	Succinate production pathway	Maximum theoretical yield (mol mol^−1^)
	PTS	The reductive branch of the TCA cycle	1
Anaerobic culture	Glucokinase	The reductive branch of the TCA cycle	1.33
	PTS	The reductive branch of the TCA cycle and activated glyoxylate pathway	1.2
	Glucokinase	The reductive branch of the TCA cycle and activated glyoxylate pathway	1.71

	PTS	The oxidative TCA cycle with inactivation of *sdhA* gene	1
Aerobic culture	Glucokinase	The oxidative TCA cycle with inactivation of *sdhA* gene and activated glyoxylate pathway	1
	PTS	The oxidative TCA cycle with inactivation of *sdhA* gene	1
	Glucokinase	The oxidative TCA cycle with inactivation of *sdhA* gene and activated glyoxylate pathway	1

These theoretical yields in anaerobic culture were calculated assuming that NADH and NAD are balanced as a result of central carbon metabolism.

These theoretical yields in aerobic culture were calculated assuming that oxygen is the H-acceptor.

**Table 3:** Coproduction of succinate and other high-value-added products using different bacteria species.

Strains	Strategies	Products	Substrates	Reference
*E. coli *KJ071	Deletion of LDH, ADHE, FOCA-PFLB, MGSA, PTA-ACKA.	33.1 g L^−1^ succinate, 69.2 g L^−1^ malate	Glucose	[8]
*E. coli* KJ073	Deletion of LDH, ADHE, FOCA-PFLB, MGSA, POXB, PTA-ACKA.	78.9 g L^−1^ succinate, 15.8 g L^−1^ malate	Glucose	[8]
*E. coli* SBS990MG	Deletion of LDH, ADHE, PTA-ACKA. Overexpression of PYC and AAT	1.22 g L^−1^ isoamyl acetate, 5.37 g L^−1^ succinate	Glucose and isoamyl alcohol	[9]
*E. coli* QZ1112	Deletion of SDHA, POXB, PTA-ACKA. Overexpression of PHBCAB	24.6 g L^−1^ succinate, 4.95 g L^−1^ polyhydroxybutyrate	Glucose	[10]
*E. coli* KNSP1	Deletion of FADR, PTAS, ATOC, FADA, SDHA, PTA-ACKA. Overexpression of PHAC1	21.07 g L^−1^ succinate, 0.54 g L^−1^ 3-hydroxyoctanoate + 3-hydroxydecanoate	Glycerol and fatty acid	[49]
*K. pneumoniae* LDH526	Deletion of LDH	102.1 g L^−1 ^ 1,3-propanediol, 13.8 g L^−1 ^succinate	Glycerol	[50]
*K. pneumoniae* CICC 10011	Enhanced CO_2_ level in the medium	77.1 g L^−1 ^ 2,3-butanediol, 28.7 g L^−1 ^succinate	Glucose	[51]
*A. succinogenes* ATCC 55618	Fractional treatment and separate utilization for succinate production.	64 g L^−1 ^succinate, glucoamylase	Wheat	[52]
